# XMP‐Net: An XAI‐Based Modified Xception Model for Recognizing Monkeypox and Other Skin Diseases

**DOI:** 10.1155/bmri/1113178

**Published:** 2026-01-08

**Authors:** Ferdib-Al-Islam, Prithvi Biswas, Partha Protim Gharami, Md. Rahatul Islam

**Affiliations:** ^1^ Department of Computer Science and Engineering, Northern University of Business and Technology, Khulna, Bangladesh; ^2^ Graduate School of Life Science and Systems Engineering, Kyushu Institute of Technology, Kitakyushu, Japan, kyutech.ac.jp

**Keywords:** explainable AI, Grad-CAM, LIME, modified Xception model, monkeypox disease, transfer learning

## Abstract

This research introduces “XMP‐Net,” a modified Xception–based deep learning architecture constructed for the categorization of skin conditions, with a particular focus on identifying monkeypox. The study recognizes skin images of four categories: normal, chickenpox, measles, and monkeypox. To enhance interpretability and foster confidence in the model′s predictions, Grad‐CAM (gradient‐weighted class activation mapping) and LIME (local interpretable model‐agnostic explanations) were employed to illustrate the model′s thinking manner. The model demonstrated impressive classification performance, attaining an accuracy of 98.33% for normal skin, 98.25% for monkeypox, 84.21% for measles, and 77.27% for chickenpox. Precision, recall, and *F*1‐score values were also analyzed for each class, with monkeypox achieving a precision of 91.80%, a recall of 98.25%, and an *F*1‐score of 94.92%. The visual explanations generated by Grad‐CAM and LIME highlighted critical parts in the input images that affected the model′s likelihoods, offering clinicians valuable insights into the diagnostic process. This research underscores the potential of explainable artificial intelligence (XAI) in augmenting traditional diagnostic methods, particularly for emerging communicable maladies like monkeypox, and provides a foundation for developing reliable, interpretable, and accessible diagnostic tools for resource‐constrained settings.

## 1. Introduction

Monkeypox, a virus‐related zoonotic disease [1], has re‐emerged as a universal health concern [2, 3], with outbreaks reported in over 100 countries and more than 87,000 confirmed cases as of 2023 [4]. Its symptoms, including skin rashes and lesions, often resemble other dermatological conditions such as chickenpox and measles, leading to challenges in accurate and timely diagnosis. Traditional investigative techniques, such as polymerase chain reaction (PCR), are operative but costly and time‐consuming and require specific laboratory facilities, which are often inaccessible in resource‐constrained regions [5]. In such settings, misdiagnosis can result in delayed treatment, contributing to the further spread of the disease and increased mortality. While machine learning and deep learning models have revealed promise in automating disease exposure, existing studies on monkeypox classification often suffer from limited datasets and a lack of explainability and focus solely on binary classification, making them inadequate for real‐world clinical scenarios. There is a pressing need for a reliable, interpretable, and cost‐effective system that can classify monkeypox and differentiate it from similar conditions to aid early detection [6] and control the disease′s spread [7].

Previous research on monkeypox classification with deep learning and machine learning reveals several significant gaps. One major limitation is the reliance on small, imbalanced datasets [8–12], which hinders the models′ generalizability and robustness across diverse populations and conditions. Additionally, many studies lack explainability [9–11, 13–15], as they function as black‐box systems [16], reducing trust and clinical applicability. A common concentration on binary classification overlooks the need for multiclass classification to distinguish between similar diseases like chickenpox or measles. Models often exhibit poor cross‐domain generalization, failing to perform well on datasets from different regions or environments. Inadequate preprocessing and data augmentation methods further limit performance, while an overemphasis on accuracy neglects other crucial metrics, which are vital for imbalanced datasets. Few studies validate their models in real‐world clinical settings, raising concerns about practical applicability. Computational inefficiency is another challenge, as many high‐performing models are resource‐intensive and unsuitable for deployment in low‐resource settings where monkeypox is prevalent. Furthermore, existing research rarely focuses on early disease detection, instead targeting fully developed lesions, and often ignores the integration of temporal or clinical data that could enhance diagnostic accuracy. Addressing these gaps is essential to developing reliable, interpretable, and scalable solutions for monkeypox detection.

With the continued rise in global monkeypox cases, the demand for rapid, scalable, and intelligent diagnostic systems has never been more urgent. In recent years, the fusion of DL and medical image analysis has revolutionized disease classification tasks, offering not just high performance but also the potential for real‐time application. Despite these advancements, real‐world deployment faces several obstacles, such as model interpretability, limited data diversity, and a lack of standardization across datasets. Moreover, the visual similarity of monkeypox with other skin diseases complicates classification tasks, especially when handled by general‐purpose convolutional neural networks (CNNs) not tailored for subtle dermatological variations. Therefore, a modified, domain‐specific architecture supported by explainability mechanisms is crucial. A carefully designed multiclass framework that can discern between overlapping skin conditions while remaining computationally efficient may play a pivotal role in reducing misdiagnosis, optimizing resource allocation, and improving patient outcomes in endemic and outbreak‐prone regions. Although recent studies, such as DenseNet201‐based model [12] and the BERSFS‐CNN framework [13], have achieved strong accuracy in monkeypox identification, these approaches face important limitations. Most state‐of‐the‐art models operate as black‐box systems, offering little or no interpretability regarding how predictions are made, which restricts their adoption in clinical practice, where trust and transparency are critical. In addition, many of these architectures are computationally heavy and resource‐intensive, making them less suitable for real‐time deployment in low‐resource or point‐of‐care settings where monkeypox outbreaks often occur. In contrast, the proposed XMP‐Net is designed as a lightweight and efficient modification of the Xception model that enables robust multiclass classification across monkeypox, chickenpox, measles, and normal cases. By integrating Grad‐CAM and LIME, the system not only produces accurate predictions but also generates visual and localized explanations of the decision‐making process. This dual focus on performance and explainability distinguishes XMP‐Net from prior works, bridging the gap between the model′s accuracy and clinical applicability.

This study focuses on developing a robust system to classify monkeypox and differentiate it from other skin conditions by categorizing skin images into four individual classes: normal, chickenpox, measles, and monkeypox. To accomplish this, a modified version of the Xception model, referred to as XMP‐Net, has been implemented as a multiclass image classifier leveraging transfer learning. Transfer learning permits the model to get assistance from pretrained weights, enabling efficient learning even with limited data. The input images undergo a series of preprocessing steps to ensure consistent quality and optimize the data for training and classification. These preprocessing steps include resizing and augmentation to boost the model′s robustness against variations in input data. After preprocessing, the images are fed into XMP‐Net, which is specifically designed to perform the classification task effectively. To ensure the interpretability and reliability of the model′s expectations, explainable artificial intelligence (XAI) techniques have been integrated into the workflow. Grad‐CAM and LIME have been employed to investigate the decision‐making manner of the modified Xception‐based CNN. Grad‐CAM creates heatmaps that visually highlight the regions of the input image most prominent in the model′s expectations, while in LIME, segments of the image are perturbed (slightly altered) to analyze their impact on predictions. These XAI techniques serve an important function in understanding the specific regions of the input images that impact considerably to the model′s classification decisions, ensuring that the system is transparent and trustworthy. By integrating these explainability tools, the proposed system not only achieves accurate classification but also provides valuable perceptions into its decision‐making process, enhancing its potential for clinical application.

The following parts of the paper have been arranged as follows: The “Literature Review” section explains previous works on monkeypox disease identification with deep learning and other methodologies and their performances. The “Proposed Method” section explains the overall approach used in the proposed work in detail. The “Result and Discussion” section discusses the findings of this study and also compares its performance with previous works. Finally, the “Conclusion” section concludes the paper by addressing future research scopes.

## 2. Literature Review

Many procedures have been suggested for the automatic identification of monkeypox disease utilizing deep learning–based computer vision and other methodologies. This section outlines the current research on the classification of monkeypox disease. Table 1 illustrates a summary of the prior research.

**Table 1:** Summary of previous research works on monkeypox disease identification.

**Author**	**Dataset size (in no. of images)**	**Method/model**	**Performance in accuracy (%)**
Sitaula and Shahi [1]	1754	Ensemble of Xception and DenseNet‐169	87.13
Ahsan et al. [8]	161	VGG‐16	78
Sathwik et al. [9]	228	VGG‐19	92
Sahin et al. [10]	228	MobileNetV2	91.11
Ali et al. [11]	228	ResNet‐50	82.96
Bala et al. [12]	228	Modified DenseNet‐201	93.19
Prabhu et al. [14]	477	Swin Transformer	89.8
Almufareh et al. [15]	477	InceptionV3	94
Uysal [16]	770	CNN+LSTM	87

Sitaula and Shahi [1] devised a transfer learning methodology by evaluating 13 distinct pretrained deep learning models, yielding mean ppv, sensitivity, *F*1‐score, and accuracy of 85.44%, 85.47%, 85.40%, and 87.13%, respectively, with their suggested ensemble technique. Glock et al. [17] used transfer learning and a deep CNN. Utilizing the ResNet‐50 model on the diverse rash image dataset, they achieved a recall of 81.7%, a specificity of 97.1%, and an accuracy of 95.2%. Ahsan et al. [8] used web mining algorithms to gather dermatological photos, which were then verified by professionals. Subsequently, they applied the VGG‐16 model to evaluate a transfer learning method, considering two strategies [8]. The first method involved categorizing the photographs into two groups of diseases, monkeypox and chickenpox, while the second involved enhancing the images. In the absence of enhanced data, the accuracy of monkeypox identification was reported at 97%; however, this accuracy decreased to 78% after the use of augmentation. Sathwik et al. [9] used patch‐based DL models and transfer learning techniques to detect the monkeypox virus early. They achieved 92% accuracy using ResNet and VGG‐19 pretrained models [18]. The dataset is sourced from Kaggle and has two classes: monkeypox and normal skin images. The classes contain 102 and 126 images, respectively, for a total of 228 images. Because of the small dataset size, they used the augmentation technique to increase the dataset size. After augmentation, the dataset size increased to 3892. Sahin et al. [10] introduced an Android app with deep learning techniques to detect the monkeypox virus using a smartphone′s camera. The system was trained using a pretrained model. The dataset from Kaggle contained 228 original images, which were increased to 3192 with the augmentation technique. Pretrained models such as MobilenetV2 and EfficientNetB0 were used to classify the images. The best accuracy was obtained from MobilenetV2, which was 91.11%.

Ali et al. [11] used three pretrained CNN architectures. A 3 × 3 convolutional filter was applied to the layers. The dataset was managed from Kaggle. The dataset had 102 monkeypox images and 126 other images. Because of the small data size, they used augmentation techniques to increase the data size for better results. Total images increased from 228 to 3192. ResNet‐50 showed the best result among all others. An accuracy of 82.96% was the best result in this study. In order to diagnose and classify monkeypox illness at an early stage, Bala et al. [12] constructed an advanced DL model employing the database called “monkeypox skin image dataset (MSID).” They applied an augmentation strategy to enhance the dataset′s picture count and introduced “MonkeyNet,” an improved DenseNet‐201‐based deep CNN model for the multiclass identification of monkeypox from skin scans. The model attained accuracy rates of 93.19% and 98.91% for multiclass categorization of novel and supplemented datasets, respectively. A reliable mobile application can be developed based on this model to assist healthcare professionals in identifying monkeypox disease early enough. This study has the prospective to shed light on an increased understanding of the diagnosis and management of monkeypox, with the option of expanding it to a larger amount of clinical data as well as skin images in future work. Khafaga et al. [13] used Al‐Biruni′s earth radius (BER) to optimize a DCNN to classify monkeypox disease. They presented a BER optimization–based stochastic fractal search (BERSFS) to optimize the DCNN layers for enhanced performance. Secondly, they performed statistical experiments using the Wilcoxon test and analysis of variance (ANOVA) to assess the suggested methodology. The proposed model achieved 98.83% accuracy. Prabhu et al. [14] proposed a different approach by using the chameleon swarm algorithm (CSA), which employs *K*‐means clustering during photo segmentation for optimal cluster selection. They used two different datasets to evaluate the proposed model from Kaggle. They managed to get 95.9% accuracy for the first dataset and 89.8% for the second. They also mention that one constraint of this research is the lack of usable data. Almufareh et al. [15] have suggested that computer vision–based approaches to identifying MPX through skin images are more innovative and safer than trivial diagnosis strategies. The method used enables deep learning algorithms to determine whether the skin lesions are MPXV‐positive or not. This study used two datasets, including the Kaggle dataset (MSID). An innovative approach to classifying with CNNs has been put forward by Eliwa et al. [19]. By optimizing the CNN model methodology, a superior AUC was obtained compared to the nonoptimized model. It is possible to make improvements in CNN representations when the GWO optimization method is used for similar tasks. An impressive 95.3% accuracy was recorded when the GWO optimizer improved its ability to distinguish classifications in the model.

The review of previous studies disclosed performance inadequacies and restricted illness identification. The principal aim of this study is to surpass previous efforts in accurately classifying utilizing XAI, with a specific emphasis on methodologies for identifying monkeypox from dermatological photos.

## 3. Proposed Method

**Figure 1:**
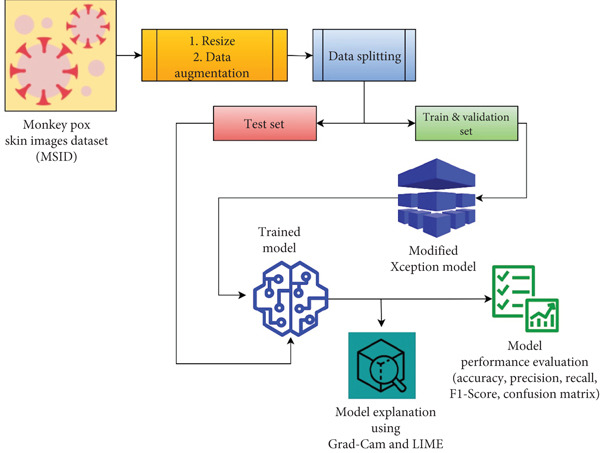
Workflow of the proposed XMP‐Net.

The approach of this research, in Figure 1, involves developing XMP‐Net, a modified Xception model, for multiclass classification of skin images. The process begins with data collection and preprocessing, where images are resized, normalized, and augmented to ensure uniformity and robustness. The preprocessed images are then fed into XMP‐Net, designed using transfer learning to leverage pretrained weights for efficient training. The model architecture is fine‐tuned to enrich its performance for the specific classification task. During training, hyperparameters are optimized. Once trained, the model′s performance is assessed using different classification metrics. To ensure interpretability, XAI techniques, specifically Grad‐CAM and LIME, are applied to the model′s predictions. These procedures provide visual and localized explanations by highlighting the regions of input images that most inform the classification decisions, ensuring transparency and reliability.

### 3.1. Dataset Description

**Figure 2:**
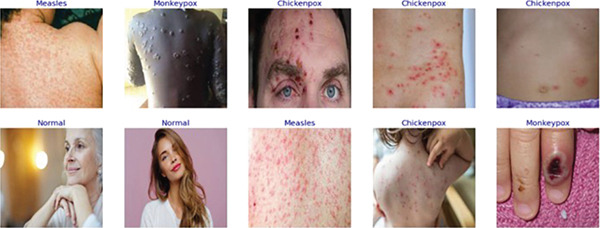
Sample images from the dataset.

The dataset we have used in this research, called “MSID,” has been collected from Mendeley Data [12, 20]. Sample images from the dataset have been illustrated in Figure 2. The dataset is divided into three partitions: training, validation, and test sets, ensuring an organized and efficient model training and evaluation process. The class‐wise dataset distribution is shown in Table 2. The training set consists of 460 images, including 175 images of monkeypox, 167 of chickenpox, 64 of measles, and 54 normal images. This partition trains the XMP‐Net model, allowing it to learn the patterns and features distinguishing the four classes. The validation set, comprising 152 images, is used to fine‐tune the model and evaluate its performance during training. This set includes 58 images of monkeypox, 55 of chickenpox, 21 of measles, and 18 normal images, helping in parameter adjustment and preventing overfitting. The test set contains 158 images and is used exclusively for final evaluation to assess the model′s generalization capability. It includes 60 images of monkeypox, 57 of chickenpox, 22 of measles, and 19 normal images. This well‐structured dataset distribution ensures a balanced representation of the classes across all partitions, enabling robust model evaluation.

**Table 2:** Class‐wise dataset size and partition.

**Partition**	**Monkeypox**	**Chickenpox**	**Measles**	**Normal**	**Total**
Training	175	167	64	54	460
Validation	58	55	21	18	152
Test	60	57	22	19	158

### 3.2. Data Preprocessing

Various preprocessing techniques were employed, including data augmentation and resizing. The entire dataset is resized to ensure uniformity in image dimensions. This method allows for the modification of system input data to avert problems resulting from data size inconsistencies. Images have been resized to 224 × 224 pixels. In our dataset, there was a noticeable class imbalance, particularly for the measles and normal categories, which contained fewer samples compared to monkeypox and chickenpox. To address this issue, we adopted multiple strategies during training. First, we applied data augmentation techniques, which helped artificially expand the underrepresented classes and increase their variability. This ensured that the model was exposed to diverse lesion appearances and reduced the risk of bias toward majority classes. Second, we incorporated class weighting in the categorical cross‐entropy loss function, allowing errors from minority classes to contribute more significantly to the overall training process, thereby balancing the influence of each class. Finally, we used a dropout layer with the dense layers of XMP‐Net, which not only improved generalization but also reduced the likelihood of the model overfitting to the majority classes. These combined measures allowed the network to learn more balanced decision boundaries and contributed to achieving reliable classification performance across all four disease categories. Table 3 presents the selected values for six transformations of image augmentation.

**Table 3:** Image augmentation parameter details.

**Parameter**	**Value**
Rotation_range	20
Shear_range	0.15
Zoom_range	0.15
Height_shift_range	0.2
Width_shift_range	0.2
Horizontal_flip	True

### 3.3. Xception‐Based CNN Architecture

**Figure 3:**
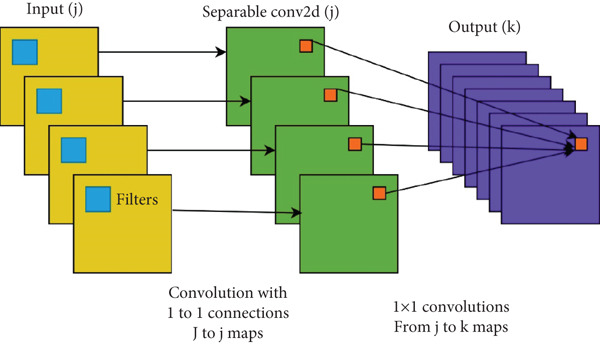
Depthwise separable convolution [23].

We chose the Xception model as the pretrained model for the XMP‐Net model because it has some advantages. It is perfect in terms of balancing accuracy with efficiency. For instance, regarding ImageNet classification tasks, Xception outperforms VGG‐16 [21] and InceptionV3 [22] in terms of accuracy, but fewer parameters are involved. With deepwise separable convolutions presented in Figure 3 included [24], architecture improvement is optimized on its part. On the contrary, XMP‐Net has mainly targeted helping feature extraction abilities associated with Xception since they are perfect for that particular purpose. ResNet‐50 is another algorithm that achieves high accuracy but at a significant cost [25]. MobileNet [26] or EfficientNet [27] could have been used under extreme resource constraints or where the main focus was either on extreme efficiency or accuracy only. However, Xception is a good compromise where accuracy and efficiency are necessary. In order to extract features from images, we need a succession of convolutional layers. Its building blocks involve small filters (generally 3 × 3) during depthwise convolutions [23] and 1 × 1 filters during pointwise ones. After this, fully connected layers interpret these features before predicting something. The first fully connected layer transforms the flattened features into a new representation using many neurons and a ReLU activation for nonlinearity. In order to prevent overfitting, regularization is employed. In its last layer, the second FCL has an output neuron for each class, and the softmax activation function, as such, generates class probabilities. This enables the model to learn complex relationships between the extracted features and the desired classifications.

### 3.4. Applying Transfer Learning

Transfer learning denotes the utilization of previously acquired skills to address a specific problem, hence improving efficiency in an alternative work. Deep learning employs several pretrained models to address specific classification challenges. The ImageNet collection has been utilized to train models that have classified 1000 categories or systems. Consequently, developing a new model by incorporating additional layers onto pretrained ones would be accomplished by this type of system training. XMP‐Net employs categorical cross‐entropy as its loss function.

**Figure 4:**
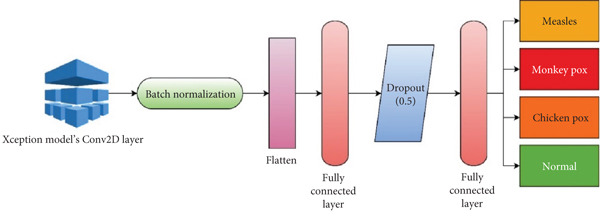
Proposed XMP‐Net.

The newly proposed CNN model utilized the essential components of the pretrained Xception model and its FCLs [28]. The weights of the Xception model were immobilized to inhibit their modification. Following the exclusion of the Xception model from the classifier, numerous new layers were integrated into the proposed CNN. Figure 4 illustrates the configuration of the suggested CNN architecture. The summary of the suggested CNN model is presented in Table 4. Initially, batch normalization was incorporated to enhance stability during training by normalizing activations. The flattened layer was incorporated to transform the 2D feature vector into a 1D feature map. The 256‐neuron fully connected layer was integrated to demonstrate the extracted features from the dataset. A dropout layer with a 0.5 threshold was used to mitigate overfitting, hence improving generalization. Finally, a second FCL was incorporated as an output layer. In the training of multiclass disease identification models, the output layer comprises four neurons.

**Table 4:** Model summary of the proposed XMP‐Net.

**Layer**	**Output size**	**Parameters**
Xception (Conv2D)	14, 14, 512	2,359,808
Batch normalization	None, 512	2048
Flatten	None, 512	0
FCL	None, 256	131,328
Dropout	None, 256	0
FCL	None, 4	1542

To minimize the risk of overfitting, several regularization techniques were applied during training. A dropout layer with a rate of 0.5 was added after the dense layer to prevent coadaptation of neurons. In addition, L2 kernel regularization was employed in the dense layer to penalize overly complex weights. We also applied data augmentation techniques, including random rotations, horizontal and vertical flips, brightness adjustments, and zooming, to increase the variability of the training data and improve generalization. Furthermore, to validate the robustness of the model, we conducted a fivefold cross‐validation (CV) experiment, where the dataset was partitioned into five subsets, and the model was trained and validated iteratively across these folds.

### 3.5. Performance Evaluation Metrics

The effectiveness of this research was assessed using multiple metrics to ensure a comprehensive assessment. Accuracy was used to measure the overall correctness of predictions, while other metrics provided a deeper analysis. Precision reflects the reliability of the model in predicting a specific class, recall indicates its ability to identify all relevant instances of that class, and the *F*1‐score balances precision and recall, offering a single measure of performance. Additionally, the confusion matrix provided a detailed visualization of the model′s predictions across all classes, highlighting specific areas of misclassification, such as between chickenpox and measles. Together, these metrics ensure a robust assessment of the models′ accuracy, reliability, and interpretability for multiclass classification tasks.

### 3.6. Decision Process Visualization Using Grad‐CAM and LIME

The decision‐making process of CNNs often functions as a black box [29], making it challenging to understand how predictions are made. To address this, visualization techniques like Grad‐CAM [30] and LIME [31] can be employed to provide insights into the model′s reasoning. Grad‐CAM produces heatmaps that highlight the regions in an input image most relevant to the CNN′s predictions. By leveraging the gradients flowing into the final convolutional layers, Grad‐CAM recognizes spatially important areas that influence the classification decision. For instance, in a skin disease classification task, Grad‐CAM can visualize which parts of an image, such as lesions or skin patterns, contribute to predicting a specific category. These heatmaps help users, including clinicians, interpret the model′s predictions and verify whether the focus aligns with medically relevant features.

LIME takes a different approach by perturbing segments of the input image and perceiving the effect on the CNN′s output. This technique identifies which specific regions of the image are critical for the classification. LIME′s explanations are model‐agnostic [32], meaning they do not depend on the internal structure of the CNN, making it versatile and easy to use with various models. In skin disease detection, LIME can provide localized explanations by highlighting individual features, such as spots or textures, that are significant for identifying a condition like monkeypox.

Combining Grad‐CAM and LIME offers a comprehensive understanding of the CNN′s decision‐making process [33], with Grad‐CAM providing a high‐level overview and LIME offering more granular insights [34]. These visualization tools not only improve model transparency but also foster trust among users, making AI‐based solutions more acceptable in sensitive applications such as healthcare [35].

## 4. Result and Discussion

Dermatological images of skin are utilized to train the multiclass CNN feature extractor in XMP‐Net. The model is constructed with the Adam optimizer, utilizing 40 epochs and a batch size of 32 for the training data. The model′s demonstrated accuracy reached saturation after 32 epochs. Early stopping and model checkpoint established the optimal epoch number. The training duration for the multiclass classifier is 25.33 min, while the test duration is 13 s.

### 4.1. Experimental Setup

The research was conducted on Google Colab, a complimentary web‐based notebook environment. Python was used for its compactness and user‐friendliness. The model was developed and trained using TensorFlow and Keras Version 2.15.0. Google Colab provided substantial resources, including 12.67 GB of RAM and 78.19 GB of disk space. To conduct model training and evaluation seamlessly, we utilized Google Drive to import data straight into Google Colab.

### 4.2. Result Analysis

This multiclass classifier achieves a validation accuracy of 93.67% and a validation loss of 0.1953. Conversely, the training accuracy is 99.89%, accompanied by a training loss of 0.1998. Figure 5 distinctly illustrates these two circumstances. The observed discrepancy between training accuracy (99.89%) and validation accuracy (93.67%) indicates a potential tendency toward overfitting. However, the application of dropout, L2 regularization, and extensive data augmentation helped mitigate this issue by improving the model′s ability to generalize to unseen data. The results of the fivefold CV further confirmed the stability of XMP‐Net, as the average accuracy, precision, recall, and *F*1‐score across the folds were consistent with the initially reported validation results. These findings suggest that, although overfitting remains a challenge in deep learning models trained on relatively small datasets, the proposed regularization and validation strategies were effective in ensuring the reliability of the classifier.

Figure 5Performance of the XMP‐Net model. (a) Training and validation accuracy and (b) training and validation loss.

**Figure:**
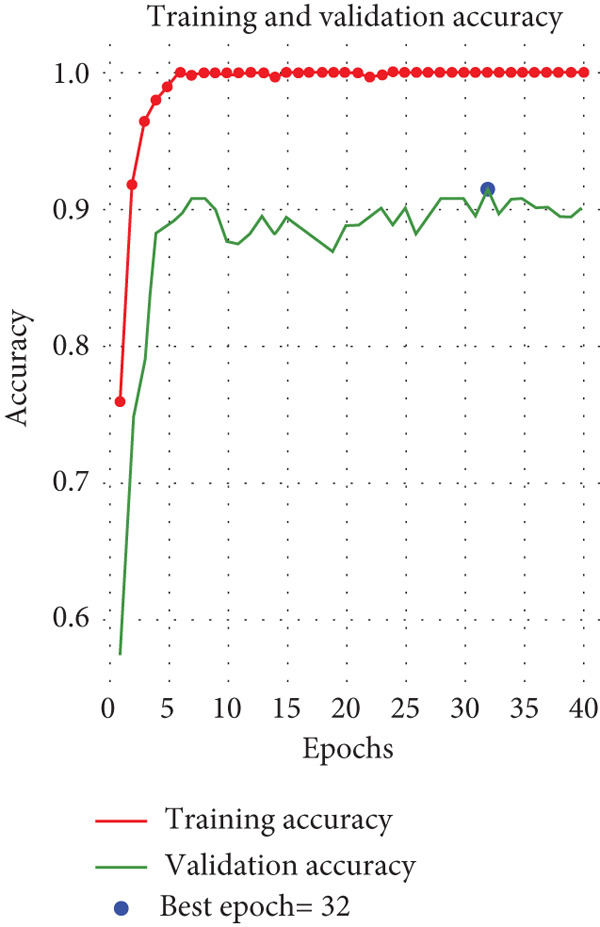
(a)

**Figure:**
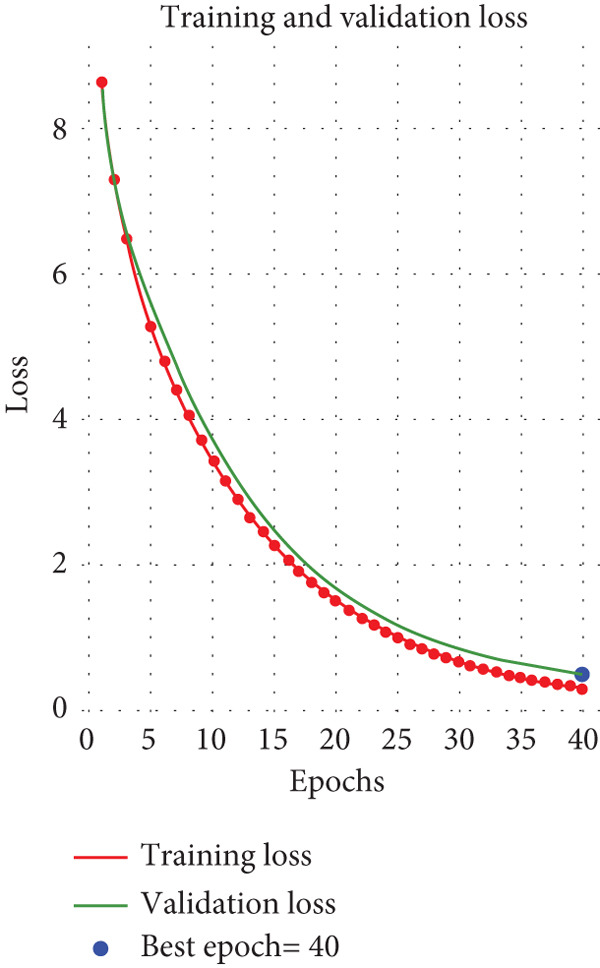
(b)

To further evaluate the robustness of XMP‐Net and reduce concerns regarding overfitting, a fivefold CV experiment was performed. In this approach, the dataset was randomly divided into five subsets, and the model was trained and validated iteratively so that each fold served once as the validation set. The results, summarized in Table 5, show that XMP‐Net consistently achieved strong performance across all folds, with an average accuracy of 93.67%, a precision of 93.61%, a recall of 93.67%, and an *F*1‐score of 93.54%. The close alignment of these metrics across different folds confirms the stability of the model and demonstrates its ability to generalize well beyond the training data. These findings reinforce the effectiveness of the applied regularization strategies and validate the reliability of XMP‐Net for multiclass skin disease classification.

**Table 5:** Performance of XMP‐Net using fivefold CV.

**Fold**	**Accuracy (%)**	**Precision (%)**	**Recall (%)**	**F**1**-score (%)**
Fold 1	93.45	93.40	93.20	93.10
Fold 2	94.10	94.10	94.30	94.00
Fold 3	92.85	93.05	93.50	93.20
Fold 4	93.72	93.80	93.90	93.80
Fold 5	94.23	93.70	93.45	93.60
Average	93.67	93.61	93.67	93.54

**Figure 6:**
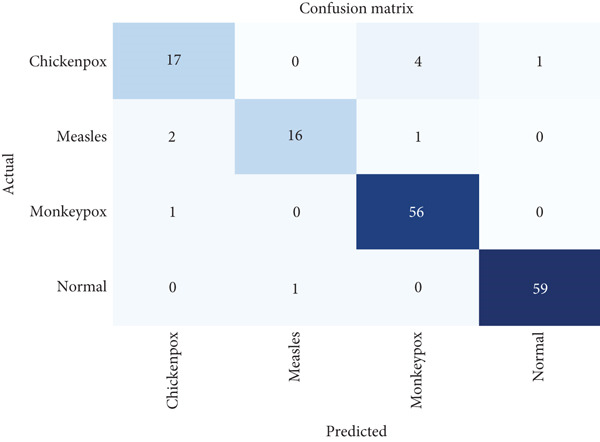
Confusion matrix of the XMP‐Net model.

The confusion matrix illustrated in Figure 6 demonstrates the classification performance of the model across four classes. For chickenpox, 17 out of 22 cases were correctly classified, with four misclassified as monkeypox and one as normal. In the measles class, 16 out of 19 cases were accurately identified, while two were misclassified as chickenpox and one as monkeypox. The model performed exceptionally well in identifying monkeypox, correctly classifying 56 out of 57 cases, with only one misclassified as chickenpox. Similarly, for the normal class, 59 out of 60 cases were accurately classified, with one misclassified as measles. Overall, the model demonstrated high accuracy for monkeypox and normal classes, while chickenpox and measles exhibited slightly higher misclassification rates, indicating room for improvement in distinguishing these conditions.

From Table 6, the performance metrics reveal that the model achieved varying levels of accuracy, precision, recall, and *F*1‐score across the four classes, highlighting its strengths and areas for improvement. For the chickenpox class, the model demonstrated moderate performance with an accuracy of 77.27% and an *F*1‐score of 80.95%, indicating some difficulty in distinguishing it from other classes. The measles class showed improved performance, with an accuracy of 84.21% and an *F*1‐score of 88.89%, suggesting better differentiation but still leaving room for enhancement. The model excelled in identifying monkeypox and normal classes, achieving high accuracy rates of 98.25% and 98.33%, respectively. These results were complemented by high *F*1‐scores of 94.92% for monkeypox and 98.33% for normal, demonstrating the model′s robustness in identifying these classes with high precision and recall. An important factor influencing the performance of XMP‐Net is the inherent class imbalance and small sample size within the MSID dataset, particularly for the measles and normal categories. The limited number of training samples in these classes restricted the model′s ability to capture diverse lesion patterns, leading to lower recall and *F*1‐scores compared to the monkeypox and chickenpox classes. Although data augmentation and class weighting strategies were employed to mitigate this imbalance, the issue could not be entirely eliminated, and some degree of bias toward the majority classes likely remained. This observation underscores the critical role of dataset diversity in training robust deep learning models for medical image analysis. Expanding the dataset with larger and more balanced class distributions would not only improve the representational capacity of the network but also enhance its generalizability across different populations and clinical settings. While the overall results highlight the model′s strong performance, particularly for monkeypox and normal cases, the relatively lower metrics for chickenpox and measles suggest potential overlaps in features that need to be addressed in future iterations of the model.

**Table 6:** Class‐wise classification report of the model.

**Class**	**Accuracy (%)**	**Overall Acc. (%)**	**Precision (%)**	**Overall Prec. (%)**	**Recall (%)**	**Overall Rec. (%)**	**F**1**-score (%)**	**Overall** **F**1 **(%)**
Chickenpox	77.27	93.67	85.00	93.61	77.27	93.67	80.95	93.54
Measles	84.21		94.12		84.21		88.89	
Monkeypox	98.25		91.80		98.25		94.92	
Normal	98.33		98.33		98.33		98.33	

**Figure 7:**
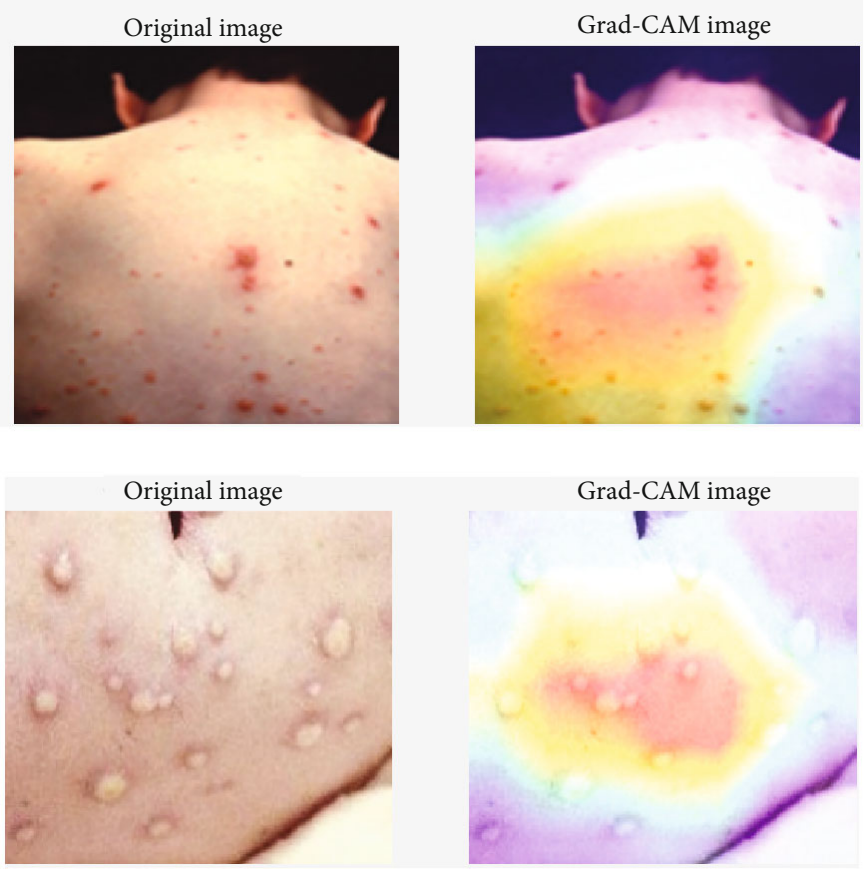
Grad‐CAM visualization.

Figure 7 shows the Grad‐CAM visualizations that effectively highlight the areas of the skin that are most influential in the model′s decision‐making process for disease classification. The heatmaps indicate that the model focuses on the central regions of the skin lesions, where the color intensity ranges from yellow to red, signifying higher relevance in the classification task. The original images show multiple skin lesions, and the Grad‐CAM overlays pinpoint these critical regions, confirming that the model accurately identifies the distinctive features of the skin conditions.

**Figure 8:**
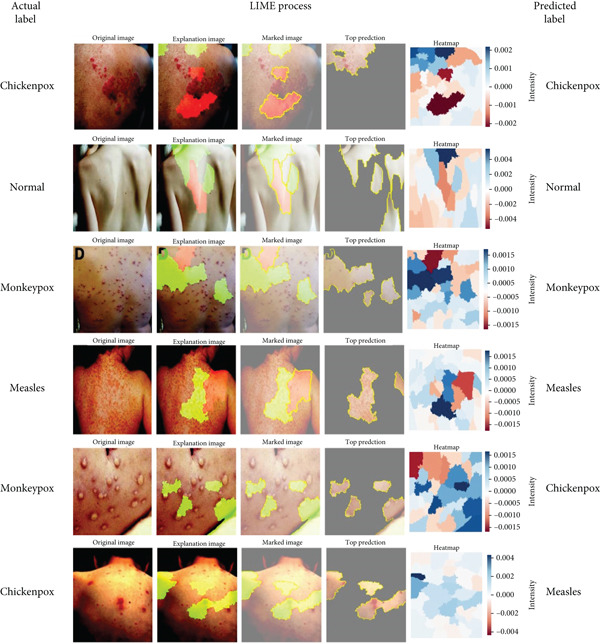
LIME visualization.

Figure 8 shows the LIME visualization. It illustrates the interpretability of the model′s decision‐making by highlighting specific regions of the skin image that contribute most to the classification outcome. The “Explanation Image” and “Marked Image” reveal areas outlined in bright green and yellow, indicating the segments that the model considered important. The “Top Prediction” section reinforces this by focusing on these marked areas, and the accompanying “Heatmap” provides a visual representation of the feature importance, with varying intensities corresponding to the impact on the prediction. This layered visualization demonstrates how LIME breaks down the model′s decision, offering a clear and interpretable insight into which parts of the image are most significant for the classification, thereby enhancing the understanding and trust in the model′s predictions. As shown in Figure 8, in correctly classified cases, such as monkeypox and normal, the highlighted regions from LIME correspond well to the actual lesion areas or lesion‐free regions, indicating that the model′s decision was based on clinically meaningful visual cues. For instance, in the correctly identified monkeypox case, LIME emphasized the vesicular lesions, which are distinct features of the disease. Similarly, in the normal class, the highlighted areas indicated lesion‐free skin regions, supporting the accurate prediction. In contrast, Figure 8 also illustrates misclassified samples. For example, one monkeypox image was incorrectly labeled as chickenpox, and the corresponding LIME explanation shows that the model′s attention was spread across both lesion and nonlesion regions, leading to feature confusion. Likewise, in a chickenpox image misclassified as measles, the heatmaps revealed that the model focused on larger skin patches rather than the vesicular lesions, which contributed to the error. These examples demonstrate how explainability maps directly reveal the regions influencing the model′s decisions, helping us interpret not only why the model is correct but also why it sometimes fails.

Table 7 provides a comprehensive comparison of the performance of our proposed work against several existing studies in skin disease classification, highlighting the effectiveness of our approach. Our modified Xception model achieved an impressive accuracy of 93.67% on a dataset of 770 images, which is higher than most of the methods cited. For instance, Ahsan et al. [8] employed VGG‐16 on a smaller dataset of 161 images, achieving a lower accuracy of 78% with LIME for model explanation. Sathwik et al. [9] and Sahin et al. [10] used VGG‐19 and MobileNetV2, respectively, on datasets of 228 images, but reported accuracies of 92% and 91.11%, without providing any model explanation. Ali et al. [11] and Uysal [16] also worked on datasets of 228 and 770 images, respectively, achieving accuracies of 82.96% with ResNet‐50 and 87% with combined CNN‐LSTM, both without model explainability. Bala et al. [12] obtained a slightly higher accuracy of 93.19% with a modified DenseNet‐201, using Grad‐CAM for visualization. However, our model surpasses Bala et al.′s [12] accuracy and further integrates both Grad‐CAM and LIME, enhancing the interpretability and transparency of the predictions. Although Khafaga et al. [13] reported a higher accuracy of 98.83% with their BERSFS‐CNN model on a dataset of 770 images, they did not provide any model explanation, which limits the clinical applicability of their findings. Our work not only achieves competitive accuracy but also emphasizes the importance of model explainability by incorporating Grad‐CAM and LIME. This dual focus on performance and interpretability positions our model as a more practical and reliable tool for skin disease diagnosis, particularly in clinical environments where understanding model decisions is crucial. The combination of a robust performance and a transparent decision‐making process makes our proposed work a significant advancement over the existing methods.

**Table 7:** Performance comparison of the proposed work with existing works.

**Work**	**Dataset size**	**Method used**	**Accuracy (%)**	**Model explanation**
Ahsan et al. [8]	161	VGG‐16	78	LIME
Sathwik et al. [9]	228	VGG‐19	92	Not found
Sahin et al. [10]	228	MobileNetV2	91.11	Not found
Ali et al. [11]	228	ResNet‐50	82.96	Not found
Bala et al. [12]	228	Modified DenseNet‐201	93.19	Grad‐CAM
Prabhu et al. [14]	477	Swin Transformer	89.8	Not found
Almufareh et al. [15]	477	InceptionV3	94	Not found
Uysal [16]	770	CNN+LSTM	87	Not found
Proposed work	770	Modified Xception	93.67	Grad‐CAM and LIME

## 5. Conclusion

This study introduces a modified Xception‐based model for the classification of monkeypox and analogous dermatological disorders, incorporating XAI approaches like Grad‐CAM and LIME to improve interpretability. The model has robust classification efficacy, especially in distinguishing between monkeypox and normal skin states, attaining elevated accuracy, precision, recall, and *F*1‐scores. This approach′s primary strength is in its capacity to deliver visual explanations, hence enhancing confidence and transparency in clinical applications. The work has notable drawbacks, including dependence on a restricted dataset, which may compromise the model′s generalizability across varied demographics and imaging settings. Moreover, the model′s comparatively diminished efficacy in recognizing chickenpox suggests potential for enhancement in managing analogous dermatological disorders. Subsequent studies ought to concentrate on augmenting the dataset to encompass a broader range of diverse and representative images, enhancing the model′s efficacy for difficult classes, and investigating lightweight architectures for implementation in resource‐limited environments. Furthermore, integrating temporal and clinical data with imaging could significantly improve diagnostic precision and relevance in practical situations. Future studies will incorporate expert consultation to strengthen the interpretability and clinical reliability of the explainability maps. In future implementations, this system could be integrated into mobile health platforms, enabling frontline healthcare workers to perform preliminary screenings using smartphone cameras. Such deployment could significantly improve early detection rates in remote or underserved regions where laboratory testing is not readily available. Additionally, incorporating this model into clinical decision‐support systems may enhance diagnostic workflows and reduce clinician burden. At the same time, ethical considerations such as safeguarding patient data privacy, ensuring informed consent, and minimizing algorithmic bias must be prioritized to promote equitable use across diverse populations. By addressing these aspects, XMP‐Net can evolve into a practical, safe, and trustworthy diagnostic support system in both clinical and community healthcare settings.

## Conflicts of Interest

The authors declare no conflicts of interest.

## Funding

No funding was received for this manuscript.

## Data Availability

The dataset used in this research is publicly available in Mendeley Data [20].

## References

[1] Sitaula C. and Shahi T. B. , Monkeypox Virus Detection Using Pre-Trained Deep Learning-Based Approaches, Journal of Medical Systems. (2022) 46, no. 11, 10.1007/s10916-022-01868-2. PMC9535233 36201085

[2] Jezek Z. , Marennikova S. S. , Mutumbo M. , Nakano J. H. , Paluku K. M. , and Szczeniowski M. , Human Monkeypox: A Study of 2,510 Contacts of 214 Patients, Journal of Infectious Diseases. (1986) 154, no. 4, 551–555, 10.1093/infdis/154.4.551, 2-s2.0-0022447023. 3018091

[3] Nolen L. D. , Osadebe L. , Katomba J. , Likofata J. , Mukadi D. , Monroe B. , Doty J. , Hughes C. M. , Kabamba J. , Malekani J. , Bomponda P. L. , Lokota J. I. , Balilo M. P. , Likafi T. , Lushima R. S. , Ilunga B. K. , Nkawa F. , Pukuta E. , Karhemere S. , Tamfum J. J. M. , Nguete B. , Wemakoy E. O. , McCollum A. M. , and Reynolds M. G. , Extended Human-to-Human Transmission During a Monkeypox Outbreak in the Democratic Republic of the Congo, Emerging Infectious Diseases. (2016) 22, no. 6, 1014–1021, 10.3201/eid2206.150579, 2-s2.0-84969150993, 27191380. 27191380 PMC4880088

[4] Kusuma I. Y. , Visnyovszki Á. , and Bahar M. A. , Mapping the Mpox Discourse: A Network and Sentiment Analysis, Exploratory Research in Clinical and Social Pharmacy. (2024) 16, 100521, 10.1016/j.rcsop.2024.100521. 39494157 PMC11530924

[5] Leendertz S. A. J. , Gogarten J. F. , Düx A. , Calvignac-Spencer S. , and Leendertz F. H. , Assessing the Evidence Supporting Fruit Bats as the Primary Reservoirs for Ebola Viruses, EcoHealth. (2015) 13, no. 1, 18–25, 10.1007/s10393-015-1053-0, 2-s2.0-84939231436. 26268210 PMC7088038

[6] Ahmadi S. , Amirzadeh M. , Ahmadi M. , and Soleiman-Meigooni S. , From Outbreaks to Artificial Intelligence: A Comprehensive Review of Monkeypox Virus Epidemiology, Diagnosis, Treatment, Vaccination, and Deep Learning Applications, Journal of Tropical Medicine. (2024) 2024, 6688914, 10.1155/jotm/6688914. 39764350 PMC11703581

[7] Malik S. , Ahmad T. , Ahsan O. , Muhammad K. , and Waheed Y. , Recent Developments in Mpox Prevention and Treatment Options, Vaccine. (2023) 11, no. 3, 10.3390/vaccines11030500. PMC10057056 36992085

[8] Ahsan M. M. , Uddin M. R. , Farjana M. , Sakib A. N. , Momin K. A. , and Luna S. A. , Image Data Collection and Implementation of Deep Learning-Based Model in Detecting Monkeypox Disease Using Modified VGG16, https://arxiv.org/abs/2206.01862.

[9] Sathwik A. S. , Naseeba B. , Kiran J. C. , Lokesh K. , Ch V. S. D. , and Challa N. P. , Early Detection of Monkeypox Skin Disease Using Patch Based DL Model and Transfer Learning Techniques, EAI Endorsed Transactions on Pervasive Health and Technology. (2023) 9, 10.4108/eetpht.9.4313.

[10] Sahin V. H. , Oztel I. , and Oztel G. Y. , Human Monkeypox Classification From Skin Lesion Images With Deep Pre-Trained Network Using Mobile Application, Journal of Medical Systems. (2022) 46, no. 11, 10.1007/s10916-022-01863-7. PMC9548428 36210365

[11] Ali S. N. , Ahmed M. T. , Paul J. , Jahan T. , Sani S. M. , Noor N. , and Hasan T. , Monkeypox Skin Lesion Detection Using Deep Learning Models: A Feasibility Study, 2022, https://arxiv.org/abs/2207.03342.

[12] Bala D. , Hossain M. S. , Hossain M. A. , Abdullah M. I. , Rahman M. M. , Manavalan B. , Gu N. , Islam M. S. , and Huang Z. , MonkeyNet: A Robust Deep Convolutional Neural Network for Monkeypox Disease Detection and Classification, Neural Networks. (2023) 161, 757–775, 10.1016/j.neunet.2023.02.022. 36848828 PMC9943560

[13] Khafaga D. S. , Ibrahim A. , el-Kenawy E. S. M. , Abdelhamid A. A. , Karim F. K. , Mirjalili S. , Khodadadi N. , Lim W. H. , Eid M. M. , and Ghoneim M. E. , An Al-Biruni Earth Radius Optimization-Based Deep Convolutional Neural Network for Classifying Monkeypox Disease, Diagnostics. (2022) 12, no. 11, 10.3390/diagnostics12112892, 36428952. PMC9689640 36428952

[14] Prabhu M. , Sathishkumar A. , Sasi G. , Yong L. C. , Shanker M. C. , and Selvakumarasamy K. , Monkeypox Detection Using CSA Based K-Means Clustering With Swin Transformer Model, Journal of Machine and Computing. (2024) 4, no. 2, 400–407, 10.53759/7669/jmc202404038.

[15] Almufareh M. F. , Tehsin S. , Humayun M. , and Kausar S. , A Transfer Learning Approach for Clinical Detection Support of Monkeypox Skin Lesions, Diagnostics. (2023) 13, no. 8, 10.3390/diagnostics13081503. PMC10137438 37189603

[16] Uysal F. , Detection of Monkeypox Disease From Human Skin Images With a Hybrid Deep Learning Model, Diagnostics. (2023) 13, no. 10, 10.3390/diagnostics13101772. PMC10217161 37238256

[17] Glock K. , Napier C. , Gary T. , Gupta V. , Gigante J. , Schaffner W. , and Wang Q. , Measles Rash Identification Using Transfer Learning and Deep Convolutional Neural Networks, Proceedings of the 2021 IEEE International Conference on Big Data (Big Data), 2021, IEEE, 3905–3910, 10.1109/bigdata52589.2021.9671333.

[18] Saha P. , Sadi M. S. , Aranya O. F. M. R. , Jahan S. , and Islam F.-A. , COV-VGX: An Automated COVID-19 Detection System Using X-Ray Images and Transfer Learning, Informatics in Medicine Unlocked. (2021) 26, 100741, 10.1016/j.imu.2021.100741. 34549079 PMC8445760

[19] Eliwa E. H. I. , Koshiry A. M. E. , El-Hafeez T. A. , and Farghaly H. M. , Utilizing Convolutional Neural Networks to Classify Monkeypox Skin Lesions, Scientific Reports. (2023) 13, no. 1, 10.1038/s41598-023-41545-z. PMC10475460 37661211

[20] Bala D. and Hossain M. S. , Monkeypox Skin Images Dataset (MSID), 2023, Mendeley Data, V6, https://data.mendeley.com/datasets/r9bfpnvyxr/6.

[21] Simonyan K. and Zisserman A. , Very Deep Convolutional Networks for Large-Scale Image Recognition, 2014, https://arxiv.org/abs/1409.1556.

[22] Rimal K. , Shah K. B. , and Jha A. K. , Advanced Multi-Class Deep Learning Convolution Neural Network Approach for Insect Pest Classification Using TensorFlow, International journal of Environmental Science and Technology. (2022) 20, no. 4, 4003–4016, 10.1007/s13762-022-04277-7.

[23] Singh K. , Gupta S. , Mohan N. , Shastri S. , Kumar S. , Mansotra V. , Sinha A. , and Khalid S. , Effective Detection of COVID-19 Using Xception Net Architecture: A Technical Investigation Using X-Ray Images, Health Informatics Journal. (2025) 31, no. 3, 14604582251363519, 10.1177/14604582251363519, 40730345. 40730345

[24] Huo H. , Yu Y. , and Liu Z. , Facial Expression Recognition Based on Improved Depthwise Separable Convolutional Network, Multimedia Tools and Applications. (2022) 82, no. 12, 18635–18652, 10.1007/s11042-022-14066-6. 36467439 PMC9686458

[25] Younesi A. , Ansari M. , Fazli M. , Ejlali A. , Shafique M. , and Henkel J. , A Comprehensive Survey of Convolutions in Deep Learning: Applications, Challenges, and Future Trends, IEEE Access. (2024) 12, 41180–41218, 10.1109/ACCESS.2024.3376441.

[26] Khan S. U. R. , Asif S. , Bilal O. , and Ali S. , Deep Hybrid Model for Mpox Disease Diagnosis From Skin Lesion Images, International Journal of Imaging Systems and Technology. (2024) 34, no. 2, 10.1002/ima.23044.

[27] Talukder M. A. , Layek M. A. , Kazi M. , Uddin M. A. , and Aryal S. , Empowering COVID-19 Detection: Optimizing Performance Through Fine-Tuned EfficientNet Deep Learning Architecture, Computers in Biology and Medicine. (2023) 168, 107789, 10.1016/j.compbiomed.2023.107789. 38042105

[28] Sathya R. , Mahesh T. R. , Bhatia Khan S. , Malibari A. A. , Asiri F. , Rehman A. , and Malwi W. A. , Employing Xception Convolutional Neural Network Through High-Precision MRI Analysis for Brain Tumor Diagnosis, Frontiers in Medicine. (2024) 11, 10.3389/fmed.2024.1487713, 39606635. PMC11601128 39606635

[29] Rasool N. , Wani N. A. , Bhat J. I. , Saharan S. , Sharma V. K. , Alsulami B. S. , Alsharif H. , and Lytras M. D. , CNN-TumorNet: Leveraging Explainability in Deep Learning for Precise Brain Tumor Diagnosis on MRI Images, Frontiers in Oncology. (2025) 15, 1554559, 10.3389/fonc.2025.1554559, 40206584. 40206584 PMC11979982

[30] Hossain S. S. , Al-Islam F. , Islam M. R. , Rahman S. , and Parvej M. S. , Autism Spectrum Disorder Identification From Facial Images Using Fine Tuned Pre-Trained Deep Learning Models and Explainable AI Techniques, Semarak International Journal of Applied Psychology. (2025) 5, no. 1, 29–53, 10.37934/sijap.5.1.2953b.

[31] Iftikhar S. , Anjum N. , Siddiqui A. B. , Rehman M. U. , and Ramzan N. , Explainable CNN for Brain Tumor Detection and Classification Through XAI Based Key Features Identification, Brain Informatics. (2025) 12, no. 1, 10.1186/s40708-025-00257-y. PMC12044100 40304860

[32] Al-Islam F. , Sanim M. S. , Goh K. O. M. , Mahmud S. M. H. , and Nandi D. , Alzheimer′s Disease Prediction Using ANOVA With t-SNE Feature Selection Techniques and Ensemble Learning, Journal of Advanced Research Design. (2025) 144, no. 1, 123–147, 10.37934/ard.144.1.123147.

[33] Can Z. and Aydin E. , Explainable CNN–Radiomics Fusion and Ensemble Learning for Multimodal Lesion Classification in Dental Radiographs, Diagnostics. (2025) 15, no. 16, 10.3390/diagnostics15161997. PMC12385016 40870848

[34] Ergün U. , Çoban T. , and Kayadibi İ. , BCECNN: An Explainable Deep Ensemble Architecture for Accurate Diagnosis of Breast Cancer, BMC Medical Informatics and Decision Making. (2025) 25, no. 1, 10.1186/s12911-025-03186-2. PMC12519638 41084046

[35] Islam M. R. , Kumar Godder T. , Ul-Ambia A. , al-Islam F. , Nag A. , Ahamed B. , Tanzim N. , and Ahmed M. E. , Ensemble Model-Based Arrhythmia Classification With Local Interpretable Model-Agnostic Explanations, IAES International Journal of Artificial Intelligence. (2025) 14, no. 3, 10.11591/ijai.v14.i3.pp2012-2025.

